# Effects of Ginsenoside Rg1 Regulating Wnt/*β*-Catenin Signaling on Neural Stem Cells to Delay Brain Senescence

**DOI:** 10.1155/2019/5010184

**Published:** 2019-12-04

**Authors:** Yue Xiang, Shun-he Wang, Lu Wang, Zi-ling Wang, Hui Yao, Lin-bo Chen, Ya-ping Wang

**Affiliations:** Laboratory of Stem Cells and Tissue Engineering, Chongqing Medical University, Chongqing 400016, China

## Abstract

This is a study on the relationship between the protective effect of ginsenoside Rg1 on senescent neural stem cells and Wnt-*β*/catenin signaling pathway. *Background*. Recent studies have shown that overactivation of the Wnt/*β*-catenin signaling pathway is closely related to stem cell senescence. Whether Rg1 delays the senescence of NSCs is related to the regulation of this signaling pathway. *Methods*. The whole brain of Nestin-GFP transgenic newborn rat was extracted, and NSCs were extracted and cultured to P3 generation. The following indicators were detected: (1) NSC culture identification, (2) the effect of LiCl on the proliferation and survival rate of NSCs, (3) the effect of ginsenoside Rg1 on the proliferation and survival of NSCs, (4) the growth of NSCs in each group observed by an optical microscope, (5) the cell cycle of each group detected by flow cytometry, (6) the proliferative ability of each group detected by BrdU, (7) the fluorescence intensity of Nestin and Sox2 of NSCs in each group observed by a fluorescence microscope, (8) the positive rate of senescence staining analyzed by SA-*β*-Gal staining, (9) the localization of *β*-catenin in NSCs observed by laser confocal microscopy, and (10) the changes of the Wnt/*β*-catenin pathway-related proteins in each group detected by Western blotting. *Results*. LiCl activates the Wnt/*β*-catenin pathway and promotes mouse neural stem cell senescence. Ginsenoside Rg1 promotes proliferation of neural stem cells and inhibits Wnt/*β*-catenin pathway activation. *Conclusions*. LiCl can activate the Wnt/*β*-catenin signaling pathway of NSCs, and ginsenoside Rg1 can antagonize the senescence of NSCs caused by activation of the Wnt/*β*-catenin signaling pathway and delay brain aging.

## 1. Introduction

With the development of modern science and technology, it has been found that stem cells are not “undead” cells. As they age, stem cells will gradually age and die. When stem cells age, their self-renewal and multidifferentiation abilities will gradually decline. Gene mutations result in heterotypic proliferation and differentiation, and as a result, the structure and function of tissues and organs gradually decrease, and the damaged tissues are difficult to repair and regenerate. During aging, many tissues show a decline in regenerative potential coupled with a loss of stem cell function. This will lead to the development of related diseases [1–4]. There is even literature that states all aging can be explained by stem cells. The rare populations of stem cells with the potential to self-renew and differentiate regulate tissue homeostasis and the regenerative capacity [5, 6]. The neural stem cells (NSCs), able to generate neurons and glial cells, reside in the subventricular zone of the lateral ventricle and the subgranular zone of the dentate gyrus of the hippocampus [7, 8]. According to studies, the regeneration ability of neural stem cells gradually declines, leading to degeneration and dysfunction of the brain tissue, and eventually causes many degenerative diseases of the central nervous system, such as Parkinson's disease and Alzheimer's disease [9, 10].

Modern medicine believes that traditional Chinese medicine ginseng has extensive pharmacological effects on the central nervous system, cardiovascular system, digestive system, and endocrine system. Ginseng is now widely used in clinical practice and planting is strengthened, but its specific efficacy is still unclear. It can increase physical and mental activity and enhance the body's nonspecific resistance to harmful stimuli [11–15]. Ginsenosides are the main pharmaceutical ingredients of ginseng, among which ginsenoside Rg1 is an antiaging active ingredient [16–19]. Our group has been studying the antiaging effect of Rg1. It is not just the nervous system. We found that Rg1 inhibits human mesenchymal stem cell and hematopoietic stem cell senescence in vitro [20–22]. Rg1 can also inhibit brain aging in mice. In our previous studies, we found that ginsenoside Rg1 treatment improved the cognitive performance of C57 mice administrated with d-gal and increased the number of NSCs in the hippocampus of brain-aged mice. Ginsenoside Rg1 promoted neurogenesis in the dentate gyrus as well as NSC differentiation into the neurons rather than glial cells in the hippocampus of brain-aged rats. It suggests that the neuroprotective effects of Rg1 on the d-gal-induced aging mice model closely relate to the protection of NSCs [23, 24].

Discovered by consulting the literature, the Wnt family of signaling proteins participates in multiple developmental events during embryogenesis and has also been implicated in adult tissue homeostasis. Wnt signals are pleiotropic, with effects that include mitogenic stimulation, cell fate specification, and differentiation. As the volume of Wnt literature is increasing rapidly, a few aspects of current interest have been selected here, mainly focused on Wnt signaling through its receptors (Frizzleds) to *β*-catenin, which is often called the canonical pathway [25–27]. Recent studies have demonstrated that the activation of the Wnt/*β*-catenin signaling pathway is closely related to stem cell aging [28, 29]. Whether ginsenoside Rg1 delays the aging of NSCs is related to the regulation of this pathway, and no relevant reports have been reported. This experiment combines the traditional theory of antiaging of the traditional Chinese medicine and the latest theory and research techniques of stem cell aging to construct an in vitro aging model of NSCs, focusing on the study of the relationship between ginsenoside Rg1 delaying the aging of NSCs and regulating Wnt/*β*-catenin signaling pathways. The prevention and treatment of degenerative diseases provide new ideas and strategies.

## 2. Results

### 2.1. Culture and Identification of Nestin-GFP Transgenic Mouse NSCs

**Figure 1:**
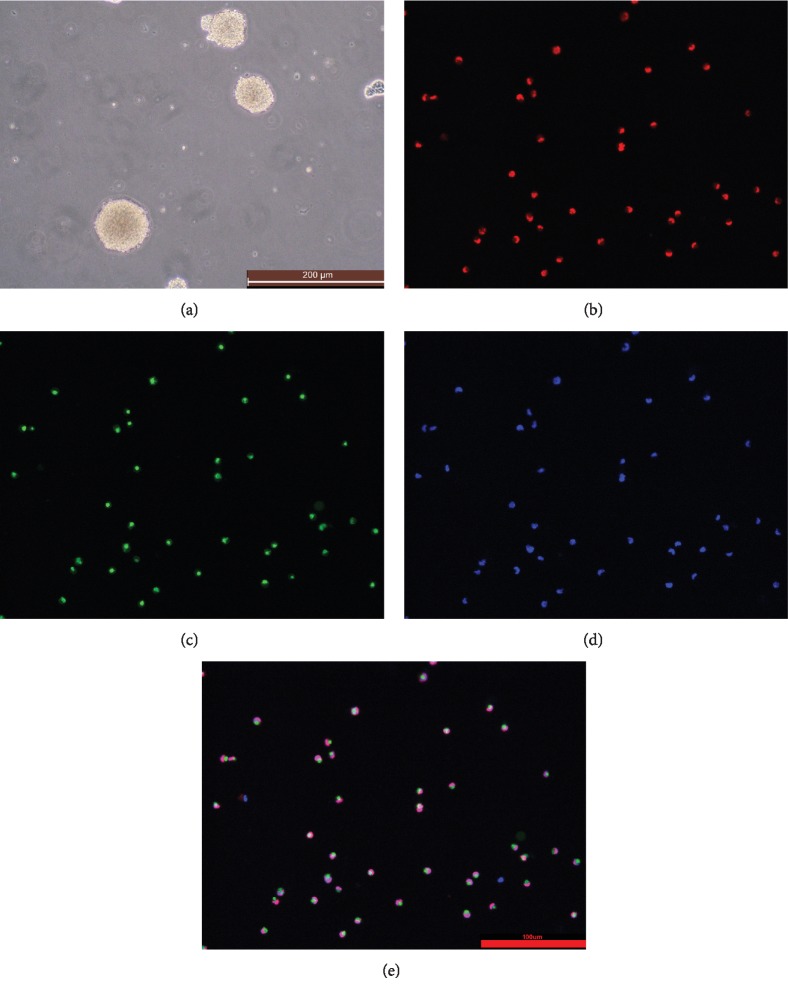
Nestin-GFP transgenic mouse NSC culture and identification: (a) primary neurosphere (×100); (b) Sox2 (×200); (c) Nestin (×200); (d) DAPI (×200); (e) merge (×200).

The brain tissue that has just been obtained is cultured in massive cell mass. The NSCs were cultured in complete medium for 10 days. NSCs formed three-dimensional structural neurospheres. Passage to P3 generation NSCs still has a good self-advanced ability, that is, 2-3 d can form new neurospheres, and the neurosphere can contain Nestin green fluorescence under a fluorescent microscope. After double labeling with Sox2 protein, the cytoplasmic portion of neural stem cells under the fluorescence microscope was stained with Nestin green fluorescence, and the nucleus portion showed Sox2 red fluorescence (Figure 1).

### 2.2. Effect of Different Concentrations of LiCl on the Survival Rate of NSCs

**Figure 2:**
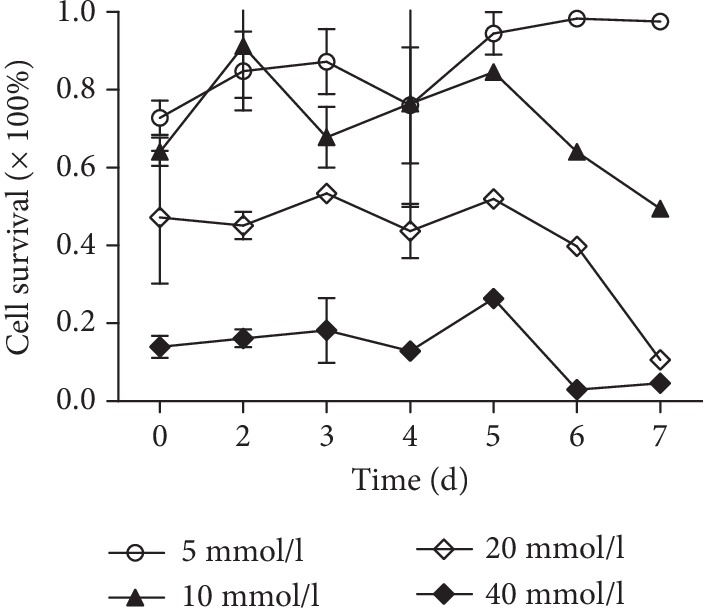
Effects of different concentrations of LiCl on the survival rate of NSCs.

After culturing in a LiCl medium of 5 mmol/l, 10 mmol/l, 20 mmol/ml, and 40 mmol/l, the CCK-8 assay method was used to detect the OD value of each group, and the cell survival rate was calculated. When the concentration of LiCl was 5 mmol/l and 10 mmol/l, the cell survival rate decreased to 0.6 (×100%) on the first day, and then, the survival rate increased again and fluctuated continuously. When the LiCl concentration was 20 mmol/l, the cell survival rate became 0.5 (×100%), and the cell survival rate fluctuated less from the first day to the fifth day. When the LiCl concentration was 40 mmol/l, the cell viability was as low as 0.1 (×100%). The subsequent experiment used LiCl concentration of 20 mmol/l, the action time was 2 d (Figure 2).

### 2.3. Effects of Different Concentrations of Ginsenoside Rg1 on the Growth of NSCs

**Figure 3:**
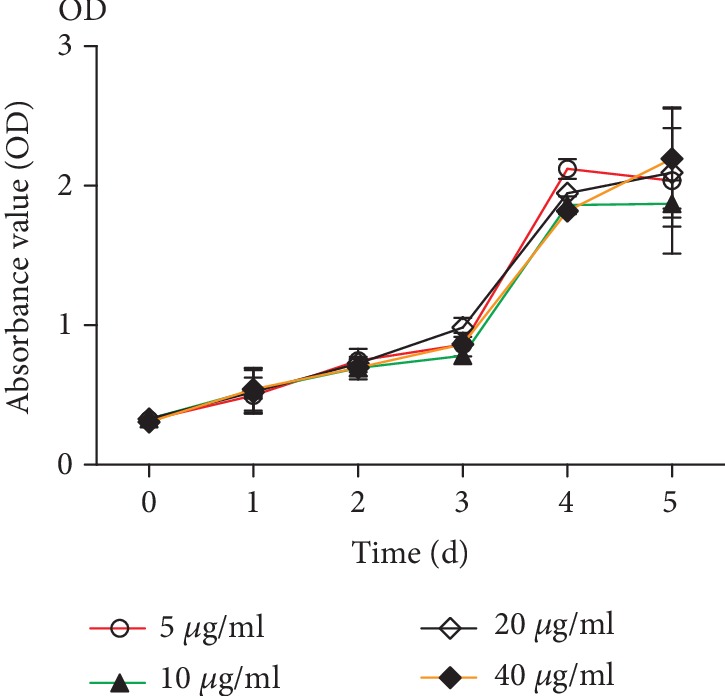
Effects of different concentrations of Rg1 on the NSC growth curve.

NSCs were cultured in different concentrations of ginsenoside Rg1 medium, and the OD value of each group was determined by the CCK-8 experimental method. There was no significant difference in OD values between each group at 1-2 d. On the 3rd day, the OD value of the 20 *μ*g/ml ginsenoside Rg1 concentration group was the highest, and the cell proliferation appeared at the earliest stage. A concentration of ginsenoside Rg1 of 20 *μ*g/ml and a dose of LiCl of 1 day were selected as subsequent experimental conditions (Figure 3).

### 2.4. Effect of Ginsenoside Rg1 on the Growth of NSCs

**Figure 4:**
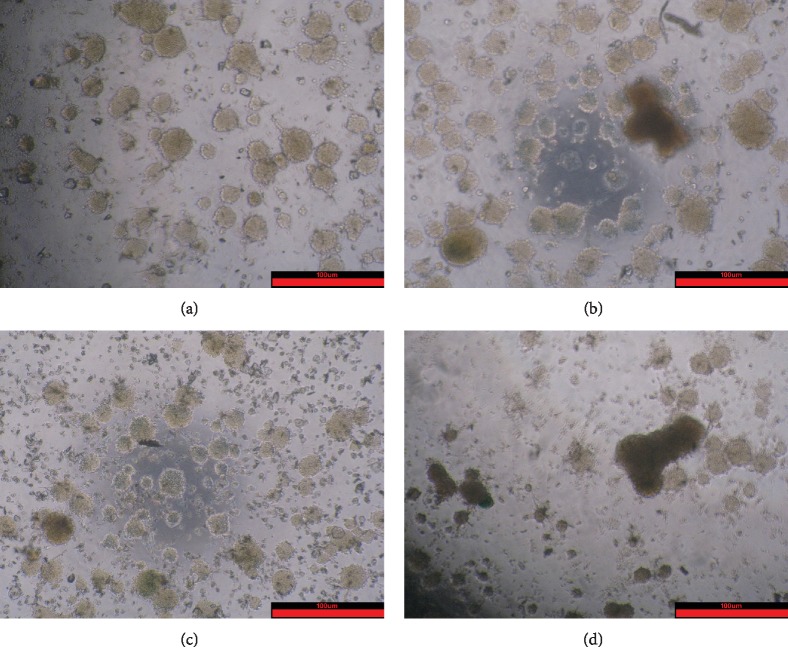
Effects of ginsenoside Rg1 on NSC growth (×200): (a) control group; (b) Rg1 group; (c) Rg1+LiCl group; (d) LiCl group.

The observation results showed that the Rg1 group formed larger and more neurospheres, and the LiCl group formed fewer neurospheres and different sizes. Compared with the LiCl group, the Rg1+LiCl group formed more neurospheres and the size was regular (Figure 4).

### 2.5. Effect of Ginsenoside Rg1 on the Cell Cycle of NSCs

**Figure 5:**
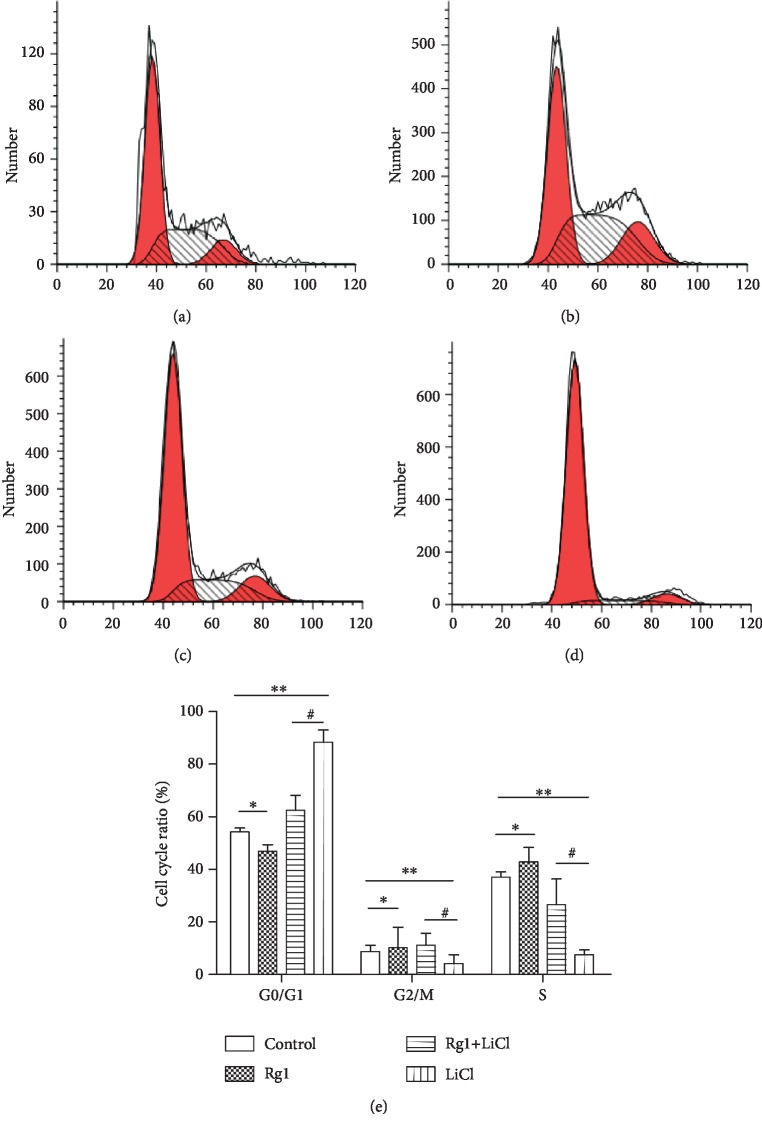
Effect of ginsenoside Rg1 on the NSC cell cycle: (a) control group; (b) Rg1 group; (c) Rg1+LiCl group; (d) LiCl group; (e) cartogram. ^∗,∗∗^Compared to the control group *P* < 0.05; ^#^compared to the LiCl group *P* < 0.05.

The results showed that the percentage of cells in the S phase and the G2 phase of the Rg1 group was significantly increased and the percentage of cells in the S phase and the G2 phase of the LiCl group was significantly decreased. Compared with that in the LiCl group, the percentage of cells in the S phase and the G2 phase of the Rg1+LiCl group was significantly increased (Figure 5).

### 2.6. Effect of Ginsenoside Rg1 on the Proliferation of NSCs

**Figure 6:**
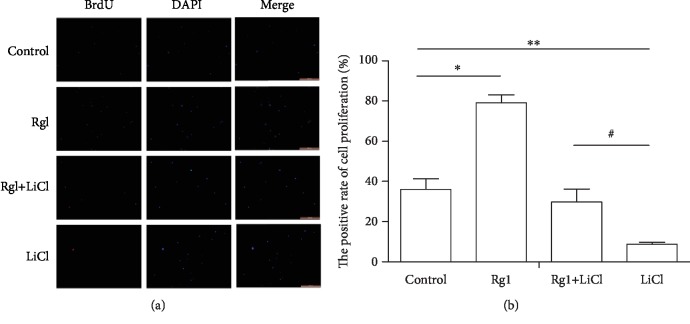
Effect of ginsenoside Rg1 on the proliferation ability of NSCs. (a) Fluorescence map of each group (×100); (b) cartogram. ^∗,∗∗^Compared to the control group *P* < 0.05; ^#^compared to the LiCl group *P* < 0.05.

The results showed that compared with the control group, the ratio of proliferating cells in the Rg1 group was significantly increased, and the ratio of proliferating cells in the LiCl group was decreased. The ratio of proliferating cells in the Rg1+LiCl group was increased compared to the LiCl group (Figure 6).

### 2.7. Effect of Ginsenoside Rg1 on the Expression of Nestin and Sox2 Protein in NSCs

**Figure 7:**
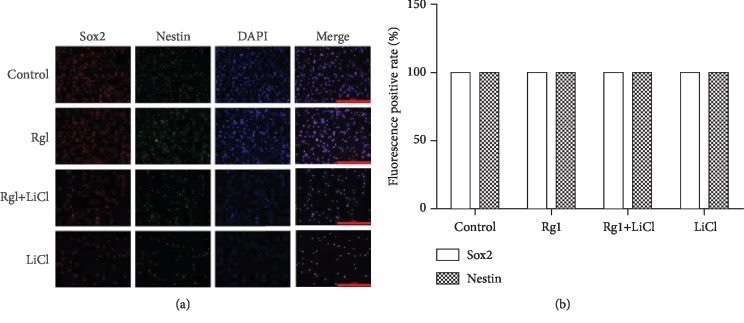
The effect of ginsenoside Rg1 on the expression of Nestin and Sox2 protein in NSCs. (a) Fluorescence map of each group (×200); (b) cartogram.

The results showed that there was no significant difference in the positive rate of Nestin and Sox2 protein in each group of NSCs (Figure 7).

### 2.8. Effect of Ginsenoside Rg1 on the Formation of Senile Neurospheres by NSCs

**Figure 8:**
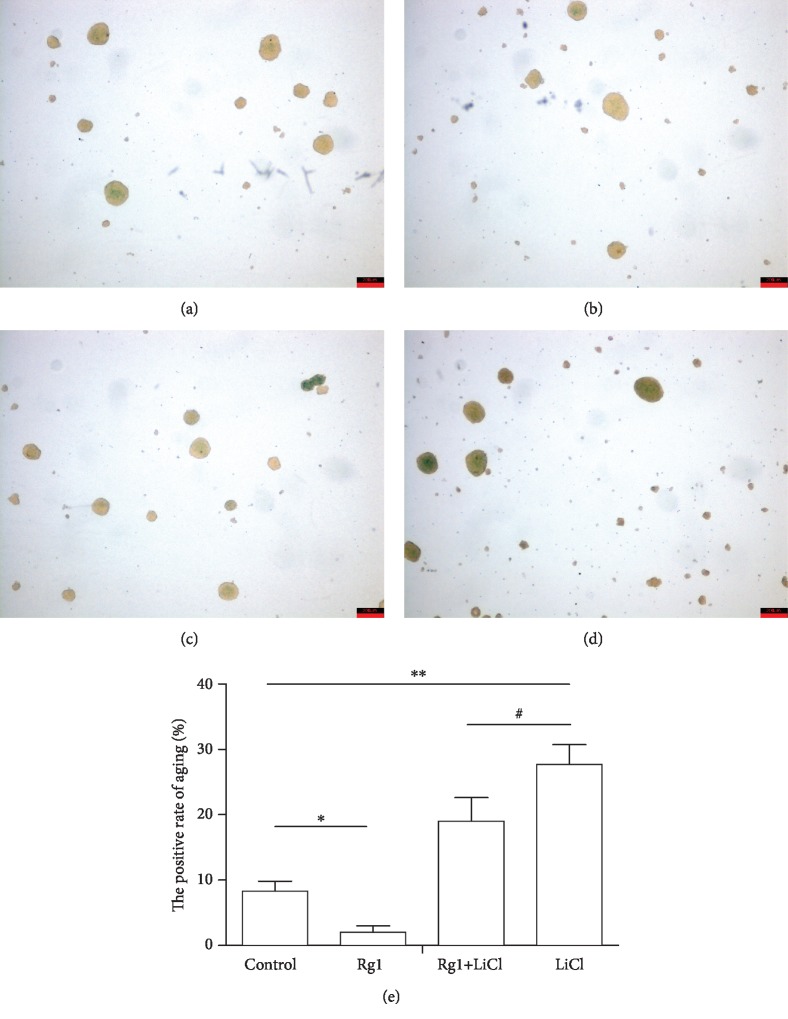
The effects of ginsenoside Rg1 on NSCs in various groups were studied (Sa-*β*-gal staining (×40)): (a) control group; (b) Rg1 group; (c) Rg1+LiCl group; (d) LiCl group; (e) cartogram. ^∗,∗∗^Compared to the control group *P* < 0.05; ^#^compared to the LiCl group *P* < 0.05.

The results showed that compared with that in the control group, the percentage of SA-*β*-Gal senescence staining in the Rg1 group was significantly decreased, and the percentage of neurospheres in the LiCl group was significantly increased. Compared with that in the LiCl group, the percentage of senile neurospheres in the Rg1+LiCl group was significantly reduced (Figure 8).

### 2.9. Effects of Ginsenoside Rg1 on Intracellular *β*-Catenin Distribution in Each Group of NSCs

**Figure 9:**
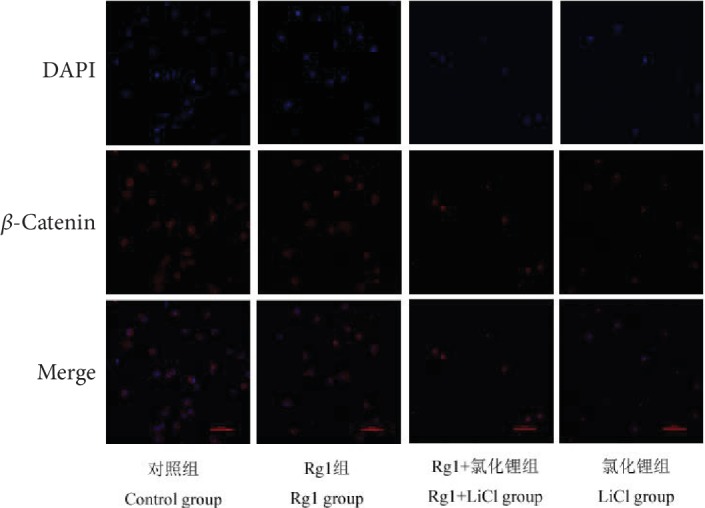
The effects of ginsenoside Rg1 on the distribution of intracellular beta-catenin in NSCs (×400).

The results showed that compared with that in the control group, the cytoplasmic *β*-catenin of the NSCs in the LiCl group was less and the number of cells was small. Compared with the LiCl group, the Rg1+LiCl group had more cytoplasmic *β*-catenin and a larger number of cells (Figure 9).

### 2.10. Effects of Ginsenoside Rg1 on the Expression of Wnt/*β*-Catenin Signaling Pathway Proteins in NSCs

**Figure 10:**
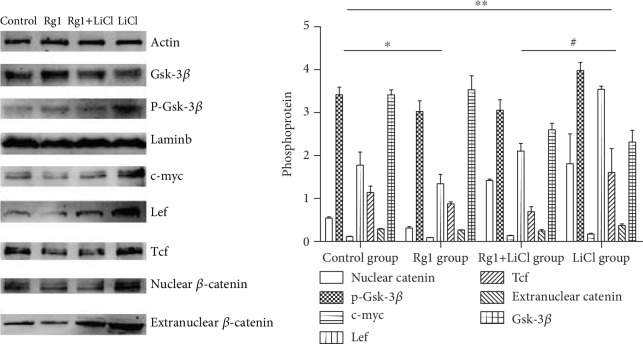
The effects of ginsenoside Rg1 on the expression of the Wnt/*β*-catenin signaling pathway in NSCs in each group. ^∗,∗∗^Compared to the control group *P* < 0.05; ^#^compared to the LiCl group *P* < 0.05.

The results showed that the nuclear catenin, extranuclear catenin, Tcf, Lef, p-Gsk-3*β*, and c-myc in the Rg1 group were decreased, and Gsk-3*β* was increased. The nuclear catenin and extranuclear catenin in the nucleus of the LiCl group were observed. Tcf, Lef, p-Gsk-3*β*, and c-myc were all increased, and Gsk-3*β* was decreased. Compared with those in the LiCl group, the nuclear catenin, extranuclear catenin, Tcf, Lef, p-Gsk-3*β*, and c-myc in the Rg1+LiCl group were decreased, and Gsk-3*β* was increased (Figure 10).

## 3. Discussion

Our previous studies have shown that brain degenerative diseases in mice are closely related to oxidative stress-induced NSC senescence and that ginsenoside Rg1 can promote hippocampal neurogenesis, improve neural plasticity, and enhance learning and memory. It has the effects of antiaging, antifatigue, and delaying brain senescence in mice. Ginsenoside Rg1 can delay brain senescence by regulating NSCs, but its specific mechanism is unclear [23, 24].

Studies have found that neuronal cell distribution and differentiation, neurodevelopment, and other changes can cause neuropsychiatric disorders and even developmental malformations. The proliferation and differentiation of NSCs is one of the main causes of neurodevelopmental disorders [30–32]. The regenerative capacity of NSCs are gradually declining, leading to tissue degeneration and dysfunction of the brain and eventually causing many degenerative diseases of the central nervous system, such as Parkinson's disease and Alzheimer's disease [33–35]. Our previous observations suggest that the neuroprotective effects of Rg1 on the d-gal-induced aging mice model might closely relate to the protection of NSCs. How to prevent and treat neurological degenerative diseases caused by aging of NSCs is a key research topic today. In this experiment, Nestin-GFP transgenic mouse whole brain was cultured in vitro to culture NSCs. It can be observed that the neurosphere structure is formed in vitro, and each neurosphere is a three-dimensional spherical structure formed by hundreds of NSCs. Nestin and Sox2 proteins are characteristic markers of NSCs. Nestin is a characteristic marker of neural stem cells, which is expressed in hippocampal dentate gyrus neural stem cells. With the differentiation and migration of neural stem cells, Nestin gradually disappears, and the proliferation and differentiation of neural stem cells can be observed by characteristic markers. Sox2 is another marker of NSCs and is commonly used for NSC identification [36]. The fluorescent staining technique was used to label Sox2 with red fluorescence. It can be observed that the cytoplasmic part of neural stem cells showed Nestin green fluorescence, and the nucleus part showed Sox2 red fluorescence. It is proved that the culture of NSCs in vitro is successful and can be used in subsequent experiments.

Recent studies have shown that the activation of the Wnt/*β*-catenin signaling pathway is closely related to stem cell senescence. LiCl is an activator of the Wnt/*β*-catenin signaling pathway [37]. The CCK-8 results showed that the cell viability decreased after the addition of LiCl. The morphology of nerve cells can be found; compared with that in the control group, the size of the neurospheres in the LiCl group is different and the shape is irregular. The ability of BrdU to detect cell proliferation was also decreased, which corresponds to the result of a significant decrease in the percentage of cells in the sodium chloride group compared with the S phase and the G2 phase of the control group. The percentage of SA-*β*-Gal senescence staining in the LiCl group was significantly higher than that in the control group. This result indicates that the in vitro aging model of NSCs was successfully established by adding LiCl.

*β*-Catenin is a key protein of Wnt signaling pathway, from the cytoplasm to the nucleus. Western blot results showed that the expression of the nuclear catenin and extracellular catenin in the nucleus of the LiCl group increased. Other proteins associated with the pathway activation Tcf, p-Gsk-3*β*, and c-myc protein in the nucleus of the LiCl group increased, and Gsk-3*β* protein decreased. It is indicated that the NSCs cultured in vitro successfully activated the Wnt/*β*-catenin signaling pathway by adding LiCl and the *β*-catenin aggregation of the NSCs in the LiCl group can be seen by laser confocal microscopy. Thus, our observations suggest that LiCl can reduce the proliferation of neural stem cells and promote cell senescence through the Wnt signaling pathway.

Modern medicine believes that Chinese medicine ginseng has a wide range of pharmacological effects on the central nervous system, cardiovascular system, digestive system, endocrine system, etc. It can improve the physical and intellectual activity and enhance the body's nonspecific resistance to harmful stimuli. Ginsenoside is the main pharmaceutical ingredient of ginseng, and ginsenoside Rg1 is an antiaging active ingredient. Our previous research has proved that brain degenerative diseases are closely related to the aging of NSCs, and the mechanism of oxidative damage is discussed. Ginsenoside Rg1 promotes hippocampal neurogenesis, improves neuroplasticity, enhances learning and memory, has antiaging and antifatigue properties, and delays brain aging in rats [23, 24]. The CCK-8 results showed that the cell growth curve increased after the addition of ginsenoside Rg1. The morphology of nerve cells can be found. Compared with that in the control group, the number of neurospheres in the Rg1 group is larger and the morphology is more regular. The ability of BrdU to detect cell proliferation was also increased, which corresponds to the results of the flow cell cycle detection of Rg1 cells compared with the control group S and G2 cells significantly increased. The percentage of SA-*β*-Gal senescence staining in the Rg1 group was lower than that in the control group. It is further proved that the effect of ginsenoside Rg1 on delaying the senescence of NSCs is consistent with the results of previous experiments. The nuclear catenin and extracellular catenin were decreased. Other activating proteins in the pathway were also reduced. This suggests that Rg1 can inhibit the aging of normal cells through the Wnt signaling pathway.

When ginsenoside Rg1 was added to the LiCl group, it can be seen that the Rg1+LiCl group formed more neurospheres than the LiCl group. Compared with that in the LiCl group, the percentage of cells in the S phase and the G2 phase of the Rg1+LiCl group was significantly increased, the fluorescence intensity of BrdU was increased, and the cell proliferation ability was increased. The percentage of senile neurospheres in the Rg1+LiCl group was significantly reduced. These observations suggest that Rg1 can regulate cell senescence induced by LiCl. Western blot showed that the expression of the nuclear catenin, extracellular catenin, Tcf, p-Gsk-3*β*, and c-myc protein in the Rg1+LiCl group decreased, and the expression of the Gsk-3*β* protein increased. It is indicated that ginsenoside Rg1 can interfere with the activation of the Wnt/*β*-catenin signaling pathway and delay the senescence of NSCs.

The results also showed that there was no significant difference in the expression of Nestin and Sox2 between the groups. This pathway does not affect Nestin or Sox 2. But the number of cells decreased. It can be seen that compared with that of the control group, the number of adherent cultures of the LiCl group was significantly reduced, but the specific reason remains to be further studied.

In summary, ginsenoside Rg1 can antagonize LiCl-induced senescence of NSCs in vitro, and its mechanism may be related to the Wnt/*β*-catenin signaling pathway. Rg1 can inhibit the activation of the Wnt/*β*-catenin signaling pathway and decrease senescence of NSCs.

## 4. Materials and Methods

### 4.1. Experimental Animals

Nestin-GFP transgenic newborn mouse (birth 1 d) was purchased from Guangzhou Saiye Biotechnology Co., Ltd. (animal certification number SYXK Yue 2008-0090), and was raised in the SPF animal laboratory dept. room of Experimental Animal Center of Chongqing Medical University. Breeding conditions are as follows: regular lighting, temperature of 20-25°C, constant humidity, air filtration, and free food and drinking water.

### 4.2. Methods

#### 4.2.1. NSC Extraction and Primary Culture

Ten newly born Nestin-GFP transgenic mice (birth 1 d) were soaked in 75% alcohol for 2 min. The heads were cut and placed in a sterile dish. Strip all brain tissue in a dish with Tissue Collection Solution. The brain tissue was rapidly smashed. Add 3 ml of the acute solution, and use a Pap tube to move the brain tissue suspension into a 15 ml centrifuge tube and mix for 5 min. Centrifuge brain tissue after 800 r/min ×5 min centrifugation, the supernatant was discarded, and 10 ml C57/BL mouse NSC complete medium was added. After mixing well, the mixture was divided into two 25 cm^2^ sterile air-permeable culture flasks, and the medium was added to make 10 ml of each flask. The medium is protected from light. Two bottles of tissue suspension were incubated in an incubator.

#### 4.2.2. NSC Exchange and Passage

Primary culture of NSCs was changed the next day, then changed every 3 days. The method of changing the liquid was as follows. Under the microscope, a large number of NSCs were found to be spherical. The tissue suspension was transferred into a 15 ml centrifuge tube using a Papman tube, centrifuge at 800 r/min for 5 minutes, discard the supernatant, add 4 ml of Accutase solution, fully blow for 5 min, centrifuge at 800 r/min for 5 minutes, discard the supernatant, add 10 ml of C57/BL mouse NSC complete medium, mix thoroughly, transfer to a 25 cm^2^ sterile air-permeable culture flask, and add the culture medium. Each bottle of 10 ml was placed in a CO_2_ incubator and then changed every 3 days. After 3 times of fluid changes, the purified NSCs can be seen under a light microscope and neurospheres formed. When some NSCs are adherent, they need to be passaged to ensure that the cell concentration of each passage is 1 × 10^5^ cells/ml.

#### 4.2.3. NSC Purification and Identification

C57/BL mouse NSC complete medium does not contain serum but contains the factors required for the proliferation of NSCs. After 3 times of exchange, only the neurospheres formed by the proliferation of NSCs remain. Nestin-GFP transgenic neonatal mice NSCs were labeled green Nestin protein fluorescence, observed under a fluorescence microscope cultured NSCs with green fluorescence. In addition, a Sox2 protein of NSCs was labeled by immunofluorescence staining. The cultivation method is as follows. 1% polylysine was immersed in sterile 20 mm pieces, and sterile slides were washed twice in PBS overnight and plated in 24-well plates. P3 NSCs that had been separated into individual cells were seeded in each well (1 × 10^5^ cells/well), incubated at 37°C for 4 h. After 4 hours, each well was washed twice with PBS, and 4% paraformaldehyde (1 ml) was added to each well to fix the cells for 30 min. Wash twice in PBS and add 1 ml of 1% Triton for 10 min. Wash twice in PBS and add 200 *μ*l goat serum to each well and shake at 37°C for 1 h. After removing the goat serum, add 150 *μ*l of Sox2 primary antibody to each well and incubate at 4°C overnight. The next day, each well was washed twice with PBS, and 200 *μ*l of a red fluorescent secondary antibody shaker was added to each well for 1 h at 37°C. Add 15 *μ*l of DAPI and shake at 37°C for 15 min. After washing with PBS for 2 times, slides were picked up and placed on glass slides with an antifluorescence quenching agent. After standing for 15 minutes, they were photographed with a fluorescence microscope.

#### 4.2.4. Effect of Different Concentrations of LiCl on NSCs

Dispensing a medium containing LiCl at a concentration of 5 mmol/l, 10 mmol/l, 20 mmol/l, and 40 mmol/l, NSCs were cultured in 96-well plates at a concentration of 5000 per well per cell for 1 d, 2 d, 3 d, 4 d, 5 d, 6 d, and 7 d in different media. The effect of different concentrations of LiCl on the viability of cells was examined with CCK-8.

#### 4.2.5. Effects of Different Concentrations of Ginsenoside Rg1 on the Proliferation of NSCs

The medium containing 5 *μ*g/ml, 10 *μ*g/ml, 20 *μ*g/ml, and 40 *μ*g/ml ginsenoside Rg1 was prepared. NSCs were cultured in 96-well plates at a concentration of 5000 per well per cell for 1 d, 2 d, 3 d, 4 d, and 5 d in different media. The effects of different concentrations of ginsenoside Rg1 on the cell growth were examined with CCK-8.

#### 4.2.6. Effects of Ginsenoside Rg1 on the Aging of NSCs Induced by LiCl In Vitro

P3 NSCs were cultured in vitro and the cells were divided into 4 groups, each with a cell concentration of 1 × 10^5^ cells/ml. Rg1+LiCl group: LiCl (20 mmol/l) was added on the first day, and ginsenoside Rg1 (20 *μ*g/ml) was added on the second day; Rg1 group was added on the first day with the same amount of medium, and ginsenoside Rg1 was added on the second day (20 *μ*g/ml); LiCl group: LiCl (20 mmol/l) was added on the first day, and the same amount of medium was added on the next day; the control group: the same amount of medium was added on the 1-2 d.

#### 4.2.7. SA-*β*-Gal Staining Detects NSC Senescence

The P3 neurospheres of each group were harvested and stained according to SA-*β*-gal staining instructions. After staining, the neurospheres were resuspended in PBS and dropped onto glass slides. The coverslips were photographed, and the number of stained positive neurospheres was counted.

#### 4.2.8. BrdU Assays the Proliferation Ability of NSCs in Each Group

Extract and purify NSCs and culture them to P3 generation (the experimental method is the same as method 4.2.1 and 4.2.2). Divide them into 4 groups (the experimental method is the same as method 4.2.6). Add BrdU to 30 mmol/l after modeling and incubate at 37°C overnight. The next day, the modeled cells were collected and washed twice with PBS. Digest and collect the cells: add 4 ml of Accutase solution and pipette well for 5 minutes, centrifuge at 1000 r/min ×10 min, and discard the supernatant. Wash each group with 500 *μ*l of washing solution twice. Fix cells: add 500 *μ*l of the fixative solution to each group and mix well at 4°C overnight. Permeabilize cells: each group was washed with 500 *μ*l washes twice, and each group was added with 500 *μ*l permeate and mixed and allowed to stand for 2 minutes on ice. Cellular DNA denaturation: the DNA denaturation working solution (300 *μ*l denaturation buffer+50 *μ*l denaturant) was stored and stored at 4°C. After washing for 30 minutes at 37°C, 500 *μ*l of washing solution was added to each group to wash twice. Label BrdU: add 195 *μ*l of staining buffer+5 *μ*l of PE-BrdU to each group and incubate for 30 min at 4°C. Label DAPI: add 10 *μ*l of DAPI to each group and incubate at room temperature for 10 minutes. Each group was washed with 500 *μ*l washes twice, photographed with a fluorescence microscope.

#### 4.2.9. Flow Cytometry to Detect the Cycle of NSCs in Each Group

Culture four groups of cells in the same way, the neurospheres were digested into single cells after completion of the modeling. The cells were washed twice with PBS. The cells were fixed at 4°C overnight with 70% alcohol, and the cell cycle of different groups was detected by flow cytometry.

#### 4.2.10. Observation of *β*-Catenin Localization in NSCs by a Laser Confocal Microscope

The 20 mm sterile slides were submerged in 1% polylysine overnight, washed twice in PBS, and dried. Plates were placed on a 24-well plate, and P3 neural stem cells that had been separated into individual cells were seeded into each well and cultured at 1 × 10^5^ cells/ml·well for 4 hours at 37°C in an incubator. After washing twice in PBS, 1 ml of 4% paraformaldehyde was added to each well for 30 minutes. After washing twice in PBS, 1 ml of 1% Triton was permeabilized for 10 minutes. After washing twice in PBS, 200 *μ*l goat serum was added to each well at 37°C for 1 hour. Remove goat serum by adding 150 *μ*l of *β*-catenin primary antibody to each well and incubate overnight at 4°C. After the second day of PBS washed twice, 200 *μ*l of red fluorescent secondary antibody was added to each well for 1 hour at 37°C. Add 15 *μ*l DAPI 37°C shaker to a secondary antibody for 15 min. Two times after washing with PBS, slides were picked up and placed on glass slides with an antifluorescence quenching agent, photographed by a laser confocal microscope after standing for 15 minutes.

#### 4.2.11. Western Blotting Detection of NSC Pathway Protein Content in Each Group

Protein samples were prepared from each group of cells, and the protein concentration was adjusted, and 40 *μ*g was added to each well. In SDS-PAGE gel separation, PVDF membrane was transferred, and 5% skim milk powder solution (1× TBST configuration) was blocked. Different antibodies (1 : 500) were incubated overnight at 4°C, goat anti-mouse secondary antibodies (1 : 3000) were incubated at 37°C for 2 h, PVDF membranes were washed, and the ECL light-emitting system developed color. ImageJ software calculated the optical density of the target protein and the internal reference protein *β*-actin value ratio. The primary antibody is configured in proportion: the ratio of nuclear *β*-catenin, extranuclear *β*-catenin, Tcf, Lef, P-Gsk-3*β*, c-myc, and Gsk-3*β* is 1 : 1000.

#### 4.2.12. Statistical Analysis

Using SPSS 20.0 statistical software, the experimental data were analyzed by one-way ANOVA. *P* < 0.05 was considered statistically significant, and the images were analyzed by IPP 6.0.

## Data Availability

The data used to support the findings of this study are included within the article.

## Abbreviations

BrdU: Bromodeoxyuridine
LiCl: Lithium chloride
Nestin-GFP: Nestin green fluorescent protein
NSCs: Neural stem cells
Rg1: Ginsenoside Rg1
SA-*β*-Gal: Senescence-associated *β*-galactosidase.
